# Choice of Steerable Sheath Impacts Contact Force Stability During Pulmonary Vein Isolation

**DOI:** 10.19102/icrm.2021.121205

**Published:** 2021-12-15

**Authors:** Evan Hiner, Dipak P. Shah

**Affiliations:** ^1^Ascension Health Providence Hospital and Medical Center, Southfield, MI, USA

**Keywords:** Ablation, atrial fibrillation, catheter stability, contact force, pulmonary vein isolation, steerable sheath

## Abstract

A stable contact force (CF) is correlated with more effective radiofrequency (RF) ablation (RFA) lesions and long-term procedural outcomes. Efforts to improve catheter stability include jet ventilation, pacing, steerable sheaths, and CF-sensing ablation catheters. This study compares CF stability and effective RF lesions between two commercially available steerable sheaths. Thirty patients underwent first-time RFA at a single center using the Agilis™ NxT (Abbott, Chicago, IL, USA) or SureFlex™ (Baylis Medical, Montreal, Canada) steerable sheath. High-power short-duration RFA was utilized, targeting a 10-Ω drop. Sheath performance was assessed for the entire procedure and around each pulmonary vein (PV) in terms of mean CF, CF variability, RF time per lesion, and inefficient contact lesions (defined as lesions with a CF of less than 5 g for at least 10% of the RF delivery time). The operator-targeted mean CF was achieved using both sheaths; however, the overall CF variability was 12.8% lower when using the SureFlex™ sheath (p = 0.08). The CF variability was generally 16% greater in the right PVs than the left PVs (p = 0.001) but trended lower with the SureFlex™ sheath. There were 8% more inefficient contact lesions created when using the Agilis™ sheath as compared to the SureFlex™ sheath (p = 0.035), especially in the right inferior PV (p = 0.009). The RF time per lesion was, on average, 12% (1.4 seconds) shorter when using the SureFlex™ sheath than the Agilis™ sheath (p < 0.05). The choice of steerable sheath may affect both catheter stability and lesion quality, especially in the right PVs.

## Introduction

While radiofrequency (RF) catheter ablation for atrial fibrillation (AF) is associated with acute pulmonary vein (PV) isolation (PVI) in over 90% of cases, long-term success of a single ablation for paroxysmal AF has been reported to be 69% at one year^1^ and only 54% beyond three years.^2^ Long-term PVI durability is often a function of continuity and transmurality of formed lesions^3^ and has been associated with the amount of RF energy delivered. The relative RF energy received by the tissue and, consequently, lesion quality are dependent on consistent coupling between the ablation catheter tip and the target tissue.^4,5^ Efforts to improve catheter–tissue contact and stability include the use of high-frequency jet ventilation to minimize respiratory excursion, cardiac pacing, electroanatomic mapping, contact force (CF)-sensing ablation catheters, and sheath selection.^6–11^ Steerable sheaths significantly enhance CF stability, facilitate mapping and ablation, and reduce procedure times when compared to fixed curve sheaths.^12^ Steerable sheaths are the active component maintaining stability during mapping and ablation compared to the passive ablation catheter. The loads these sheaths face are a function of left atrial (LA) dwell time and position. If steerable sheaths respond to these loads differently, the response to RF ablation (RFA) could be affected. In this study, CF stability and effective RF lesions were compared between two different commercially available steerable sheaths.

## Materials and methods

### Study design

A retrospective analysis was performed on 30 consecutive RFA procedures conducted at a single center by a single operator (D. P. S.) between February and June 2019. Patients with a history of previous ablation or cardiac surgery, as well as cardiac implants, were excluded from the series. An initial 15 consecutive patients undergoing PVI procedures using the 8.5-French (Fr), 72.5-cm, bidirectional Agilis™ NxT Steerable Introducer (Abbott, Chicago, IL, USA) were compared to a subsequent 15 consecutive patients undergoing PVI procedures using the 8.5-Fr, 72.5-cm, bidirectional SureFlex™ Steerable Guiding Sheath (Baylis Medical, Montreal, Canada). Standard informed consent was obtained prior to each procedure. Institutional approval was obtained for the retrospective chart review.

### Radiofrequency ablation procedure

Procedures were performed under general anesthesia and as per the usual protocol. Percutaneous access was obtained from the right and left femoral veins for all catheters. Intravenous heparin was administered to maintain an activated clotting time of approximately 350 seconds. Transseptal puncture was performed under intracardiac echocardiography and fluoroscopy guidance using the NRG Transseptal Needle (Baylis Medical) with the TorFlex Transseptal Guiding Sheath (Baylis Medical) or the SL-1 Transseptal Guiding Introducer (Abbott, Chicago, IL, USA). Three-dimensional electroanatomic mapping (EnSite Precision™ mapping system; Abbott) was used for catheter guidance and CF measurement. The Agilis™ NxT or SureFlex™ steerable sheath was used to position the ablation catheter (TactiCath Contact Force Ablation Catheter; Abbott) for point-by-point circumferential ablation using a high-power short-duration protocol of approximately 50 W RF energy. A target CF of 10 g to 15 g was applied until an impedance drop of approximately 10 Ω and a lesion size index of 4 to 6 were achieved in both sheath groups. The left superior and inferior PVs (LSPV and LIPV, respectively) were isolated before attempting to isolate the right superior and inferior PVs (RSPV and RIPV, respectively). The esophageal temperature was monitored during the ablation to remain below approximately 38°C. Acute PV reconnection was assessed by monitoring electrocardiograms, using a PV catheter (Advisor™ HD-Grid; Abbott) for electrical isolation and voltage maps for re-appearance of PV potentials within a waiting time of approximately 10 minutes.

### Data analysis

**Figure 1:**
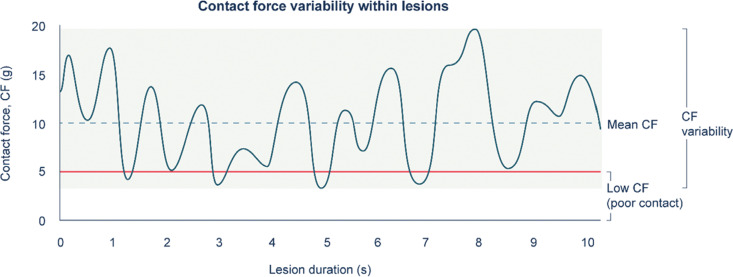
Analysis of CF parameters. Small inadvertent movements contribute to catheter instability and fluctuations in CF. Measurements obtained from the electroanatomic mapping system were used to assess mean CF and CF variability for each lesion. Poor contact was considered when CF dropped below 5 g; lesions with poor contact for more than 10% of the RF application time were defined as “inefficient contact lesions.” CF: contact force.

CF was recorded using the EnSite mapping system at approximately 10-ms intervals during each episode of RF application and was exported from the mapping system for further analysis. To account for procedural complexity, number of lesions, and overall procedure time between patients in each sheath group, CF parameters were evaluated on a per-lesion basis. The RF time per lesion and mean CF per lesion were used to assess the procedural efficiency. Stability in CF was evaluated in terms of CF variability within an individual lesion; standard deviation was calculated for the mean CF within each lesion **(Figure 1)**. Inefficient contact lesions were defined as those whereby CF dropped below 5 g (minimum level to ensure catheter–tissue contact^12^) for at least 10% of the total RF application time for the lesion.^13,14^ Analyses were performed for the overall procedure, as well as by correlating each lesion to individual PVs on the electroanatomic map.

### Statistical analysis

Baseline patient characteristics were compared using a T-test between the two steerable sheath groups. A hierarchical regression analysis was performed for the procedural parameters (mean CF, RF time per lesion, CF variability) using a linear mixed-effects model by adjusting for individual lesions and each patient in the R software (version 1.1; R Foundation for Statistical Computing, Vienna, Austria). Time-sequence data to determine inefficient contact lesions were analyzed using MATLAB (version 9.4; MathWorks, Natick, MA, USA); significance was evaluated using an F-test on R.

## Results

### Contact force analysis by location

**Figure 2:**
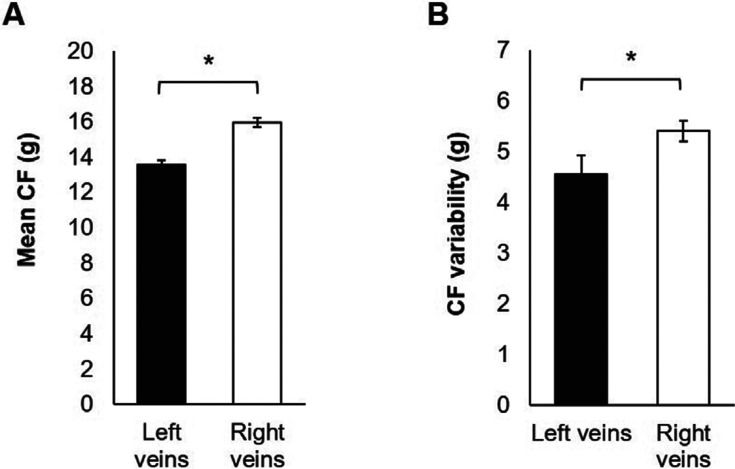
Comparison of CF in the left versus right PVs. **A:** A higher mean CF per lesion was achieved among the right-sided PVs (ie, RIPV and RSPV) than among the left-sided PVs (ie, LIPV and LSPV). **B:** A higher level of CF variability was observed among individual lesions in the right-sided veins than in the left-sided veins. CF: contact force. *p = 0.001.

A total of 3,157 lesions were analyzed with a mean of 105.2 ± 33.1 lesions per patient. Overall, the mean CF achieved during the ablation of right-sided PVs was 17% higher (p = 0.001; **Figure 2A**) than for the left-sided PVs. The CF variability within each lesion was 19% higher in the right-sided veins than in the left-sided veins (p = 0.001; **Figure 2B**).

### Contact force analysis by steerable sheath

**Figure 3:**
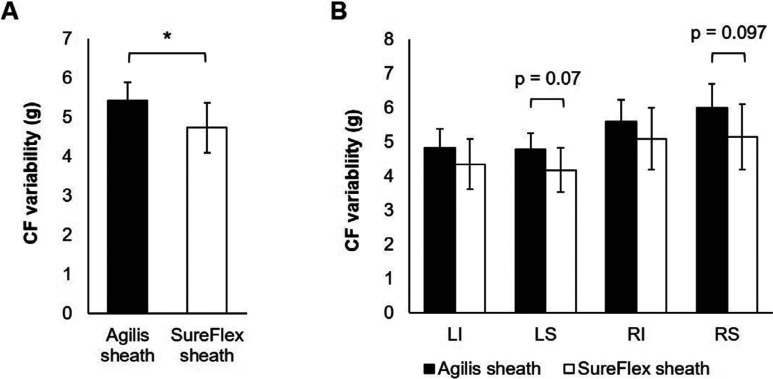
Analysis of CF variability within individual ablation lesions. **A:** A significantly lower CF variability was found when using the SureFlex™ sheath than the Agilis™ sheath. **B:** Similar trends were observed in each of the PVs with a lower CF variability when using the SureFlex™ sheath; however, this did not reach statistical significance. *p = 0.043.

Baseline patient characteristics were similar in both sheath groups except for a significantly higher body mass index (BMI) (p = 0.01) and female population (p = 0.03) in the SureFlex™ group than in the Agilis™ group **(Table 1)**. A total of 1,354 lesions were analyzed in the Agilis™ group and 1,803 lesions in the SureFlex™ group. There was no significant difference in the mean CF between the Agilis™ sheath and the SureFlex™ sheath at the overall procedure level, as well as around each PV. However, the CF variability was 13% lower with the SureFlex™ sheath over the entire procedure compared to the Agilis™ sheath (p = 0.043; **Figure 3A**). Further analysis showed a trend of 13% to 14% lower CF variability in the LSPV and RSPV with the SureFlex™ sheath than the Agilis™ sheath (significant at the α = 0.1 level; **Figure 3B**).

### Inefficient contact lesions

**Figure 4:**
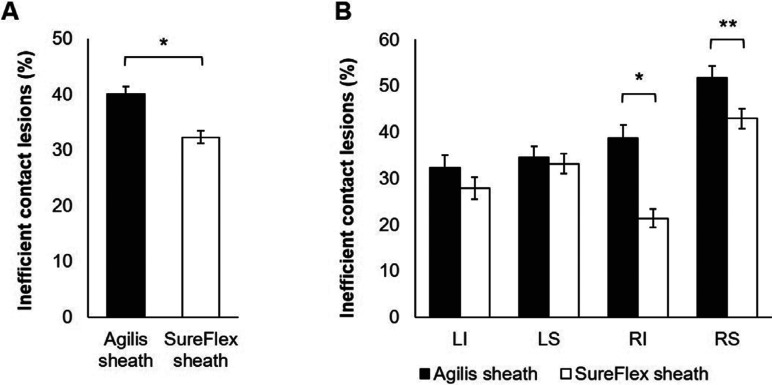
Inefficient contact lesions with greater than 10% of the ablation time below the minimum acceptable CF (CF < 5 g). **A:** Fewer inefficient contact lesions were found in the SureFlex™ group than in the Agilis™ group. **B:** Difficult-to-access right-sided PVs (ie, RIPV and RSPV) had the greatest difference in inefficient contact lesions between the SureFlex™ and Agilis™ groups. CF: contact force. *p < 0.001; **p = 0.009.

The SureFlex™ sheath maintained better catheter–tissue contact than the Agilis™ sheath, as demonstrated by 20% overall fewer inefficient contact lesions (ie, lesions with a CF of less than 5 g for more than 10% of the ablation time; p < 0.001; **Figure 4A**). The odds ratio (OR) for inefficient contact lesions was 0.605 for SureFlex™ versus Agilis™ [95% confidence interval (CI): 0.371–0.976; p = 0.035], revealing a 39% lower chance of inefficient contact lesions with the SureFlex™ sheath than the Agilis™ sheath. While this reduction was consistent among all PVs, the difference between the SureFlex™ and Agilis™ sheaths was significant in the right PVs, with 17% and 45% fewer inefficient contact lesions in the RIPV and RSPV, respectively (p < 0.01; **Figure 4B**). The OR for the SureFlex™ sheath versus the Agilis™ sheath for RIPV was 0.607 (95% CI: 0.35–1.03; p = 0.009) and that for RSPV was 0.583 (95% CI: 0.27–1.24; p = 0.15).

### Radiofrequency time per lesion

**Figure 5:**
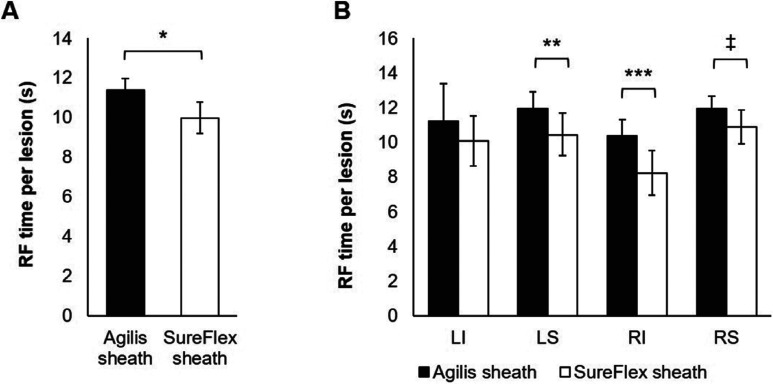
RF time per lesion. **A:** The RFA time to reach the acute lesion endpoint (ie, an impedance drop of approximately 10 Ω) was 12% shorter in the SureFlex™ group than in the Agilis™ group. **B:** The RF time per lesion was 9% to 21% lower in the LSPV, RIPV, and RSPV when using the SureFlex™ sheath. LI: left inferior; LS: left superior; RI: right inferior; RS: right superior. *p = 0.002; **p = 0.022; ***p = 0.003; ^‡^p = 0.048.

The overall RFA time per lesion was 12% (1.4 seconds per lesion) shorter with the SureFlex™ sheath when compared to the Agilis™ sheath (p = 0.002; **Figure 5A**). A 9% to 21% reduction in RFA time was observed in the LSPV, RIPV, and RSPV when using the SureFlex™ sheath compared to the Agilis™ sheath (p < 0.05; **Figure 5B**).

### Procedural outcomes

PVI was successfully achieved in all 30 patients with paroxysmal (57%) and persistent (37%) AF. No major procedure-related adverse events, such as cardiac tamponade, stroke, or esophageal injury, occurred.

## Discussion

Several steerable sheaths are commercially available but their relative influence on CF and catheter stability remains unknown. In this study, a retrospective evaluation of data from 30 patients who underwent PVI procedures using two different steerable sheaths indicated that procedural efficiency measures such as CF stability and RF time per lesion may be impacted by the choice of sheath. A consistent ablation strategy was used whereby PVs were ablated in sequence, with the left veins before the right veins, suggesting that any observed trends are attributed to both anatomy and/or sheath fatigue over time. The overall CF achieved during the ablation of the right-sided PVs was higher than the CF achieved for the left-sided PVs. Greater CF variability reached statistical significance between the right PVs and left PVs, but this difference trended lower when using the SureFlex™ sheath than the Agilis™ sheath. Both steerable sheaths in this study achieved a similar mean CF, ruling out potential procedural bias. Further, despite more extensive operator experience with the Agilis™ sheath than the SureFlex™ sheath prior to this case series, the SureFlex™ sheath maintained an overall 13% greater CF stability than the Agilis™ sheath, with similar trends in individual PVs.

Maintaining a minimum CF throughout the RF application is necessary for effective lesions and improving ablation outcomes,^13,14^ whereby insufficient tissue CF has been correlated with inadequate lesion formation^15–17^ and a higher rate of AF recurrence.^13,18,19^ The SureFlex™ sheath led to significantly fewer lesions with poor contact (< 5 g for more than 80% of the ablation time). This trend was further pronounced in the RIPV and RSPV, which are typically more difficult to navigate. Reduced CF stability may necessitate longer RFA to reach the desired lesion endpoint, potentially reducing the efficiency and increasing the risk of coagulum formation and steam pops.^5,16,20,21^ Although the total number of ablation lesions was greater in the SureFlex™ group, the RF time was assessed per lesion, targeting the same 10-Ω impedance drop. To account for the difference in ablation lesions, statistical analysis using a mixed-effects model was used. The RF time per lesion was 12% shorter in the SureFlex™ group than in the Agilis™ group, suggesting improved RF delivery to achieve the desired lesion endpoint.

To the best of our knowledge, this is the first study that compares the procedural performance of two commercially available sheaths with the goal of improving CF stability for more effective RFAs. Both the SureFlex™ and Agilis™ sheaths are bidirectional steerable sheaths, with comparable outer and inner diameters, 180° deflection clockwise, and 90° deflection counterclockwise. However, proprietary differences between these braided sheath shafts could theoretically lead to more controlled steering as well as less sheath fatigue with the SureFlex™ sheath over the duration of the procedure, thereby resulting in differences in CF variability and effective RF lesions observed in this study.

### Limitations

This single-operator nonrandomized study retrospectively evaluated the procedural parameters in a small patient population, which limited the statistical power of the analyses. While a consecutive series of patients undergoing first-time ablation was selected, the BMI and the number of women were higher in the SureFlex™ group, which may have introduced procedural complexity and technical challenges. Although baseline left atrium echo parameters were comparable between the two groups, a larger percentage of women and potentially a thinner left atrial wall could have resulted in the shorter RF duration seen with SureFlex™. The operator was not blinded to the sheath being used; however, the same method of circumferential point-by-point ablation by the same operator attempts to mitigate variability between the groups. Also, a similar mean CF was achieved using both sheaths, suggesting a lack of operator bias. Future studies assessing CF stability and effective RF lesions with other steerable sheaths as well as fixed curve sheaths and their correlation to long-term clinical outcomes will be important to explore.

## Conclusion

This study demonstrates that the choice of steerable sheath may affect CF variability and the effectiveness of RFA lesions.

## Figures and Tables

**Table 1:** Baseline Patient Demographics

Characteristics	SureFlex™ (n = 15)	Agilis™ (n = 15)	p-value
Age, average, years	60.2 ± 11.9	64.7 ± 11.0	0.30
Male sex, %	40	80	0.025
BMI, average	35.9 ± 8.8	28.8 ± 3.6	0.01
Hypertension, %	80	67	0.43
Diabetes, %	0	13	0.16
ASCVD, %	40	67	0.15
Heart failure, %	20	40	0.25
Pacemaker, %	13	0	0.16
Paroxysmal/persistent AF, %	60/27	53/47	0.72/0.27
Ejection fraction ≤ 50%, %	21	25	0.84
LVH, %	33	47	0.47
LA size—volume/BSA, average, cm^2^	38.7 ± 18.7	39.8 ± 17.9	0.89
Mitral regurgitation, %	73	67	0.70

AF: atrial fibrillation; ASCVD: atherosclerotic cardiovascular disease; BMI: body mass index; BSA: body surface area; LA: left atrial; LVH: left ventricular hypertrophy.
